# Annotations

**Published:** 1888-03-03

**Authors:** 


### Annotations.

**Bricks or Daisies?**

Miss Octavia Hill is one of those who stand, broom in hand, striving to check the incoming waves of the sea. But she is no Mrs. Partington! She and her like point out with clear logic and convincing fact the dangers and necessities of the times; and by steadfast courage and unwavering resolution bring the community to understand that a sea-wall is wanted to resist some of the surging waves of our civilisation, and that such a wall must be built forthwith. Her present mission is to prevent London from being poisoned with bad air, and to preserve for its poorer inhabitants the possibility of an occasional sight of the green turf, the trees, and the flowers. The ubiquitous builder threatens to "civilise" every square yard of field or garden within a radius of ten miles from Charing Cross. Miss Octavia Hill dares him to carry out his ignorant and health-destroying purpose; and calls upon all men and women who have feeling and sense to come to her aid. In western London there is preserved for public use, within the four-mile radius, one acre to every 682 persons; whilst in eastern London, within the four-mile radius, there is only one acre for every 7,481 inhabitants. In other words, western London has more than ten times the open space possessed by eastern London. But, says Miss Hill, "Remember that your western population . . . takes flight in July to country estate or Switzerland; that after an illness the children can mostly go to the sea . . . that they have airy bed-rooms, and day nurseries, and that the streets round them are cleaner, and the closed squares give air to their neighbourhood. Then cast your eye on the eastern semi-circle. . . . Few squares you see, and the majority of houses are let in floors or couples of rooms. Fancy a working-woman carrying a baby from, say Hoxton, to the nearest park on a hot summer day. . . . Picture the clergyman or over-worked clerk wanting a refreshing walk from Spitalfields, what vistas of streets to pass through, and always more of them as the suburban fields get covered. Yet these are the people who stay in town most, if not all of the year, whose rooms are crowded, and whose facilities for getting about are least." Miss Octavia Hill and her friends are a valiant army. If we can give them no more, we can at least express to them our gratitude and earnest hope that they will march on undaunted, whatever be the difficulties of the way or the enemies who may resist. We will also ask our readers to think what they may find it possible for themselves to do in this urgent, and in every way beneficial, public work. The poor who most need open spaces can do practically nothing for themselves. The rich and the leisured can do much; and each should make sure that he does not do less than his own proper share. It is individual men and women who do the world's work and bring it to completion.

**Doctors in Cotton Wadding.**

Medical Men are doubtless deeply grateful to certain journals which are devoted to their interests and protection—although those journals do make the profession look a little ridiculous sometimes. A touching instance of this grandmotherly fussiness appears in last week's *Lancet*. *Punch*, with a defective taste, which the *Lancet* gently deplores, has "noticed," though happily only in a "humorous" way, a "medical work," written by a "medical man," and has made "an injudicious laudatory allusion to another member of our profession." Now *Punch*, with such an experienced editor as Mr. Burnand, ought to have known better. Of course, a mere *layman* like one of *Punch's* Philistine contributors, could not be expected to be familiar with the sublime ethics and the transcendental rules of etiquette which obtain at a purely medical newspaper office, and which are dispensed from week to week with a judgment which cannot be impeached and a disinterested liberality beyond all praise. The "Lay Press," a phrase which we do not profess to understand, but which must mean something, since it is frequently used by the most distinguished medical philologists, ought to be informed that doctors are a peculiar class of men, who live and think and act and hope and fear in a world apart from other men. They are a kind of "esoteric Buddhists," whose misfortune it is to have "bodies, parts, and passions" like mortals, but whose minds are occupied with soaring thoughts and imaginings which the common literary herd can by no means comprehend or attain to. It is true their daily business is with such vulgar things as the stomachs, livers, lungs, legs, and arms of their fellow-beings. And it is equally true that those fellow-beings, finding their stomachs and livers often very troublesome, have a not unnatural desire to understand intelligently why these organs should be so worrying and unmanageable. But that is the care of the doctor, and of the doctor exclusively; and no man should dare to pry with impertinent curiosity into the mysteries of his own anatomy, physiology, therapeutics, and so on, without the doctor's permission; and no doctor ought to give any particular patient or friend such permission without first making appeal to the highest seat and centre of medical authority and enlightenment. That centre is not now, as it used to be, the General Medical Council; neither is it the College of Physicians or that of Surgeons. These two latter august bodies, it would seem, have abdicated their judicial functions in favour of government by private journalism. Be that as it may, it is expected soon to be an established rule that any doctor who writes a book or newspaper article shall, immediately on its publication, be folded up, along with his book or article, in cotton wadding, and carefully put away in a *Museum of Medical Curiosities* to be shortly erected by the liberality of private journalists. The curator of the museum will be selected on account of his merits as a teacher of ethical science in general, and of medical ethics in particular. He will have absolute control over the museum and its contents; and any person wishing to see any particular doctor or his published work will only be able to do so by obtaining the curator's permission. It is hoped that this scheme, which has long been incubating, may produce a body of medical literature whose authors no patient will wish to consult, and whose works nobody will desire to read. If nothing else is gained by the scheme, its promoters believe that purity of motive will at least be secured. This they regard as an object for which no sacrifice can be too great.

**"Home Colonisation."**

Under this somewhat paradoxical cognomen a society has been established by the Rev. Herbert Mills, an enthusiastic but eminently practical North-country clergyman. It is attracting a good deal of attention among those who have a little more sense and a little more courage than their neighbours. Several public meetings have been held in different parts of London, with the object of making the association known to those who are prepared to put their hands to the plough of social improvement, and not to look back. What does Mr. Mills want to do? And why does he want to do it? He wants to find profitable work for those who cannot find any for themselves. And he wants to do it, primarily, because as an Fnglishman and a Christian he can by no means be content or happy while he sees thousands of his fellow countrymen unable to get enough, even of plain bread, for three full meals a day; but secondarily, because he knows the kind of stuff Englishmen are made of, and that if they are condemned to starvation for a sufficient length of time, they in their turn will spring the mine of revolution upon us. A stomach unsatisfied three times a day is apt to breed, not only a wolf's appetite, but a wolf's temper. The logic of the wolf is teeth—a very unpleasant kind of logic for sheep whose syllogisms cannot be reduced to an immediately practical form. Mr. Mills knows very well, from experience among the working-classes, that they will endure a good deal, especially if they see other classes sympathising with them and trying to help them. But they will not endure for ever. Anarchy has a dreadful sound to a man who has ten thousand or even five hundred a-year; but what does a man care for anarchy who has no bread and no clean sheets, and no bed to put them on even if the sheets were in the linen-chest? He cannot be worse off than he is. He may be better off. He has nothing to lose, but his neighbours have; and if a general scramble should take place some of his neighbours' superabundant resources might fall to him. Mr. Mills wants to prevent this, and to found an agricultural and generally industrial colony, which will give employment and supply food and all other necessaries for 500 mixed people. There are several examples of this kind of colony in existence, and they are in many cases self-supporting, and, in some highly prosperous. The society has its offices at 29, Great Portland Street, W., and Mr. W. Adams is its Honorary Secretary.

